# Detecting Corresponding Vertex Pairs between Planar Tessellation Datasets with Agglomerative Hierarchical Cell-Set Matching

**DOI:** 10.1371/journal.pone.0157913

**Published:** 2016-06-27

**Authors:** Yong Huh, Kiyun Yu, Woojin Park

**Affiliations:** 1 Spatial Information Research Institute, Korea Cadastral Surveying Corp., Seoul, Korea; 2 Department of Civil and Environmental Engineering, Seoul National Univ., Seoul, Korea; Fondazione Edmund Mach, Research and Innovation Centre, ITALY

## Abstract

This paper proposes a method to detect corresponding vertex pairs between planar tessellation datasets. Applying an agglomerative hierarchical co-clustering, the method finds geometrically corresponding cell-set pairs from which corresponding vertex pairs are detected. Then, the map transformation is performed with the vertex pairs. Since these pairs are independently detected for each corresponding cell-set pairs, the method presents improved matching performance regardless of locally uneven positional discrepancies between dataset. The proposed method was applied to complicated synthetic cell datasets assumed as a cadastral map and a topographical map, and showed an improved result with the F-measures of 0.84 comparing to a previous matching method with the F-measure of 0.48.

## Introduction

Map conflation of spatial datasets from different mapping agencies usually encounters locally uneven positional discrepancies between corresponding objects of the datasets. To address these discrepancies, corresponding point pairs are necessary to align one dataset with another. In general, given a point in one dataset, several candidate points in another dataset within a distance threshold are evaluated with similarity measures such as distance, and a single point with the highest similarity is chosen as the corresponding point [1]. However, these similarities are easily affected by the aforementioned discrepancies. Thus, a transformation model, such as an affine or a rigid model, is applied to explain the locally auto-correlated positional discrepancies of each corresponding polygon object pair [1–5].

To find the above object pairs, intersection analysis has been applied which works well when the objects to be matched are sufficiently large and isolated each other within each dataset such as building objects. This is because the positional discrepancies do not significantly affect the objects’ intersection relations. Meanwhile, when the datasets are planar tessellations, the above analysis presents many erroneous intersections between cells of each tessellation. This is because the cells are mutually exclusive and collectively exhaustive, thus a cell in one dataset can significantly co-intersect cells in another dataset which represent different real-world entities [4]. Moreover, if the datasets are constructed by different mapping agencies with their own representation rules, there needs to find M:N corresponding cell-set pairs (CCPs). Considering the aforementioned erroneous intersections and complicated M:N corresponding cell-set pairs, detecting CCP with a conventional object intersection analysis is not suitable for planar tessellation datasets. Thus detecting corresponding vertex pairs (CVPs) also becomes a complicated problem.

To address the above problem, we apply the idea of object intersection-based agglomerative hierarchical co-clustering [6]. It represented polygon objects of two datasets and their intersection degrees as the nodes and edge weights of a bipartite graph, respectively, and searched object clusters by agglomerative hierarchical clustering of the nodes according to the edge weights. Then, a candidate object-set pair is obtained by dividing one object cluster into two object-sets according to the datasets to which the objects belong. Among these object-set pairs, the pairs whose shape similarities are larger than a threshold are chosen for corresponding object-set pairs. The above agglomerative clustering and evaluation approach is similar to the buffer growing algorithm of [7] which iteratively expands an edge-set pair by one segment from either of two networks until a corresponding edge-set pair is obtained. By applying this clustering analysis to cells of planar tessellation datasets, each tessellation is divided into cell-sets, and then CVPs are independently detected from each CCP. Moreover, due to the clustering property, CCPs are obtained in a hierarchical structure. Thus, given a CCP, its super-CCP which is made by merging the CCP’s neighbouring CCPs, can be also used to detect CVPs. However, initial cell intersection degrees are affected by the discrepancies. To address this problem, the above CVPs detection and map transformation with the CVPs are iterated until a termination condition. Through these iterated matching and transformation processes, the locally uneven positional discrepancies can be gradually reduced, and thus the datasets are aligned. Then final CVPs are obtained as nearest vertex pairs within a tolerance distance.

## Related Works

Many studies proposed two-phase approaches which detects corresponding object pairs, and then separately detects corresponding object pairs from each object pair with point set matching. Gösseln and Sester [3] and Butenuth et al. [2] applied the ICP algorithm to vertices extracted from contours of corresponding objects. Recently, Huh et al. [1,4] and Wang et al. [5] applied string matching methods to the contours instead of point set matching. Because of separate corresponding point pair detections for corresponding object pairs, these methods can be robust to locally uneven positional discrepancies between datasets [1].

Most of the above methods assumed 1:1 corresponding object pairs and a few studies proposed methods to detect M:N corresponding object-set pairs. Bel Hadj Ali [8] proposed a graph-connectivity-based method to integrate building datasets. He represented building objects and their intersection relationships between the datasets as nodes and edges of a bipartite graph, respectively. Through connectivity analysis of the nodes, the object clusters and their corresponding building object-set pairs can be obtained. However, this method cannot resolve erroneous intersections caused by the positional discrepancies. To address this problem, Bel Hadj Ali [8] applied a post-processing which repeatedly removes or adds one polygon object to a corresponding object-set pairs until the highest shape similarity is obtained. Meanwhile, Huh et al. [4] applied an indeterminate boundary model. Similar to Bel Hadj Ali [8], they connected the nodes only when the interior objects of original objects intersect each other so that object intersections can be robust to the discrepancies within a tolerance distance.

However, these methods are not proper for planar tessellation datasets. In case of CCPs with many small cells, the post-processing of Bel Hadj Ali [8] suffers from computational expense. Moreover, under the condition of locally uneven positional discrepancies between datasets, these small cells in one dataset can co-intersect substantially different cell-sets in another dataset and present erroneous large CCPs [1].

## Proposed Method

**Fig 1:**
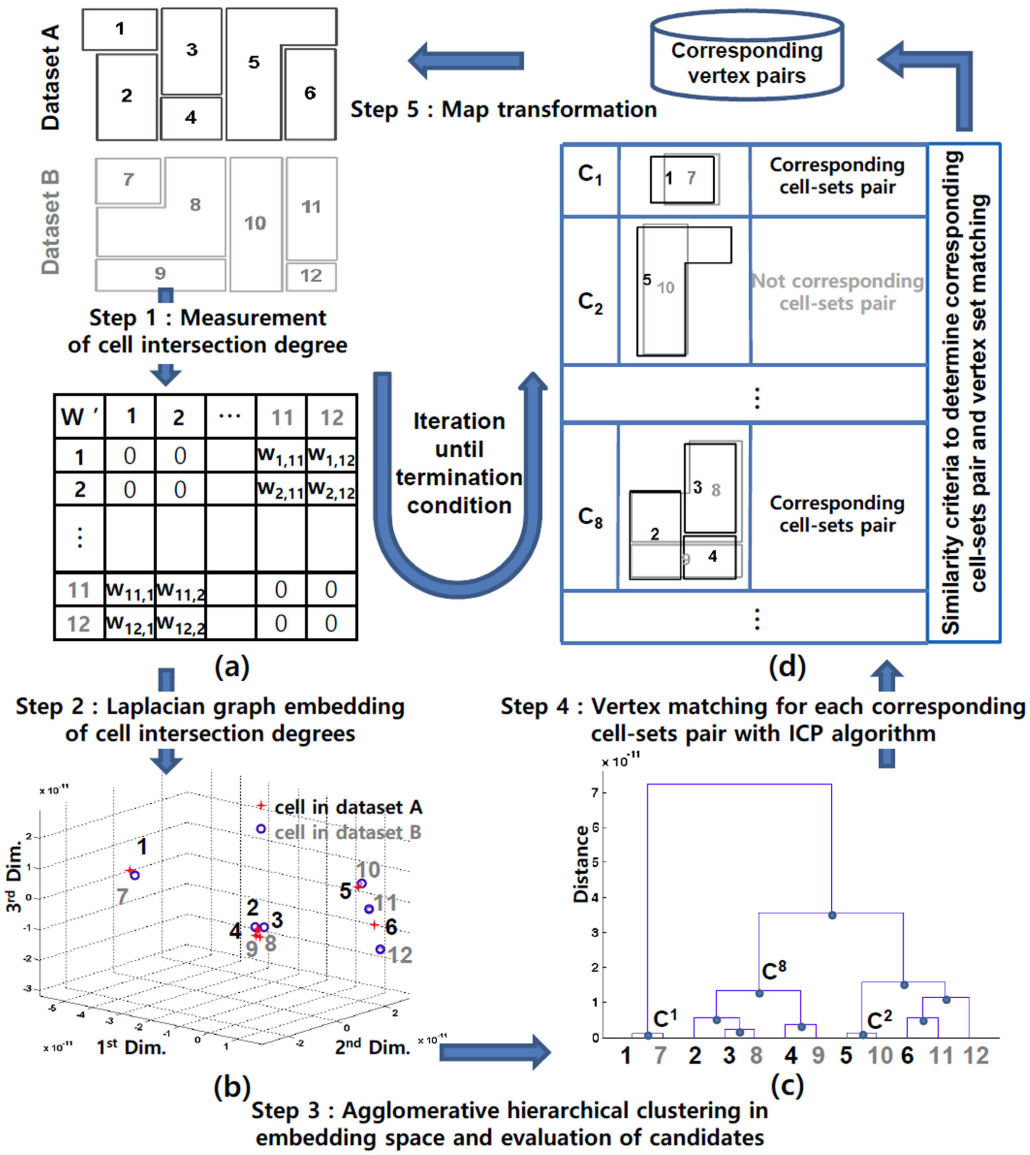
The five steps of the proposed method to find corresponding cell-set pairs and their corresponding vertex pairs between dataset A and B. The proposed method co-clusters cells of planar tessellation datasets according to the cells’ intersection degrees and evaluates cell clusters whether they are geometrically corresponding cell-set pairs. Then corresponding vertex pairs are detected from these cell-set pairs and used for map transformation to reduce positional discrepancies between datasets. These matching and transformation processes are iterated until a termination condition.

In this study, CCPs between two planar tessellation datasets are found with agglomerative hierarchical co-clustering and CVPs are detected for each CCP as shown in Fig 1. Comparing to the previous methods of Bel Hadj Ali [8] and Huh et al. [4] which treated the cell intersections between datasets as Boolean relations of 0 or 1, intersection degrees between 0 and 1 are applied (Step 1 in Fig 1). Then, the proposed method converts the cell intersection degrees into object proximities in a geometric space using a Laplacian graph embedding technique [6] (Step 2 in Fig 1). This is similar to the multidimensional scaling analysis of Mardia et al. [9] which estimated the two dimensional spatial configuration of some British cities’ locations from the road distances between the cities. Meanwhile, in this study, the cells that intersect each other with higher degrees have closer coordinates and those with lower degrees have more distant coordinates. With the coordinates of the embedded cells, cell clusters can be identified with a conventional agglomerative hierarchical clustering method (Step 3 in Fig 1). Then, each of the cell clusters is divided into two cell-sets according to the datasets to which the cells belong and evaluated with a matching criterion. CVPs for each CCP are detected with the ICP algorithm (Step 4 in Fig 1). However, the cell intersection degrees are affected by the positional discrepancies problem. To address this problem, the above CVP detection and a map transformation with the CVPs (Step 5 in Fig 1) are iterated until a termination condition. Because the datasets are gradually aligned though the iteration, final CVPs are obtained as nearest vertex pairs within a tolerance distance.

The detailed steps are presented in the following sections.

### Measurement of cell intersection degree

To detect non-1:1 CCPs which stand for 1:N CCPs and M:N CCPs, it is necessary to find the part-and-whole relationships of two cells between datasets [10, 11]. After a pre-processing to align the coordinate systems of datasets, the degree of this relationship is measured by Eq (1) as the ratio of the intersection area between cells of two datasets to the area of a smaller cell.

$$w_{i,j}=\frac{Area\;{\left(a_{i}\;\cap \;b_{j}\right)}^{}}{\text{min }\left(Area\;\left(a_{i}\;\right)\;,\;Area\;\left(b_{j}\right)\right)}\;$$ (1)

where *a*_*i*_ and *b*_*j*_ represent cells in datasets A and B, respectively.

These measures are represented by as a matrix *W* that has a size of *n*×*m*, where *n* and *m* are the number of cells in datasets A and B, respectively. Mathematically, the following Laplacian graph embedding technique assumes a symmetrical matrix of input data. However, the cell intersection degrees are measured between datasets A and B with *n* and *m* number of cells, respectively. Thus the matrix *W* of size of *n*×*m* is alternatively represented as Eq (2) to satisfy the assumption as shown in Fig 1(a) [12].

$$W\;\prime \;=\;\left[\begin{array}{cc} \begin{array}{c} 0\\ W^{T} \end{array} & \begin{array}{c} W\\ 0 \end{array} \end{array}\right]$$ (2)

### Laplacian graph embedding of cell intersection degree

Given a cell intersection degree *w*_*k*,*l*_ in *W*′, the *d*-dimensional coordinate vectors of cells *x*_*k*_ and *x*_*l*_ are obtained through the minimization of Eq (3)’s left term [13,14]. The minimization means that the cells with higher intersection degrees have close coordinates, whereas those with lower intersection degrees have distant coordinates as shown in Fig 1b. Therefore, clustering analysis of these coordinate vectors presents coherently co-intersected cell clusters between datasets.

$$\frac{1}{2}\;\sum_{k=1}^{N}\sum_{l=1}^{N}{\left\|x_{k}-x_{l}\right\|}^{\;2}\;w_{k,l}\;=\;trace\;\left(X^{T}LX\right)$$ (3)

where *N* is the total number of cells (*N* = *n* + *m*), *L* is the Laplacian matrix of *W*′ such that *L* = *D* − *W*′, *D* is the diagonal matrix such that *D*(*k*,*k*) = ∑_*k*≠*l*_*W*′(*k*,*l*) and *X* is the coordinate matrix such that [*x*^(1)^|⋯|*x*^(d)^]. Here, the entries of column vector *x*^(*p*)^ are coordinates of cells in the p^th^ dimensional space such that *x*^(*p*)^ = [*x*_1_^(*p*)^,⋯,*x*_*N*_^(*p*)^]^*T*^. Thus, *x*_*k*_ corresponds to the k^th^ row of *X* because *x*_*k*_^(*p*)^ is the coordinate of the k^th^ cell in the p^th^ dimensional space.

The left term of Eq (3) can be represented as the matrix formulation of right term of Eq (3). Thus the solution to the minimization problem {*x*^(*p*)^|*p* = 1,⋯*d*} is obtained by the eigenvectors of *L X* = *λ X* corresponding to the eigenvalue {*λ*^(*p*)^|*p* = 1,⋯*d*} under the condition 0 = *λ*^(0)^<*λ*^(1)^≤⋯≤*λ*^(*p*)^≤⋯≤*λ*^(*d*)^ [13,14].

However, the eigenproblem assumes a constraint of *X*^*T*^ *X* = *I* [14], which results in normalised coordinate vectors *x*^(*p*)^ in each dimensional space. Thus, the eigenvectors need to be scaled according to each dimension’s relative importance. Huh et al. [6] simply determined the coordinate vector of a cell as corresponding row of *X* according to Dhillon [15]. However, importance of each embedding space are not same each other [16]. Sameh and Wisniewski [17] proved that the minimum value of *trace*(*X*^*T*^*LX*) equals the sum of the eigenvalues as shown in Eq (4).

$$\text{min }trace\;\left(X^{T}L\;X\right)\;=\;\sum_{p=1}^{d}\lambda^{\;(p)}$$ (4)

Due to Eq (4), we treat *λ*^(*p*)^ as the amount of error variance in the p^th^ dimensional embedding space. Thus, in clustering, it is appropriate to apply more weight to $\left|\;x_{\;k}^{\left(p\right)}\;-\;x_{l}^{\left(p\right)}\;\right|$ than to $\left|\;x_{\;k}^{\left(p+1\right)}\;-\;x_{l}^{\left(p+1\right)}\;\right|$ because *λ*^(*p*)^ ≤ *λ*^(*p*+1)^. Therefore, in this study, *x*_*k*_ is determined as k^th^ row of *X*′ in Eq (5) [16].

$$X\;\prime \;=\;\left[\frac{x^{\left(1\right)}}{\sqrt{\lambda_{1}}},\;\cdots ,\frac{x^{\left(d\right)}}{\sqrt{\lambda_{d}}}\right]$$ (5)

where *X*′ is a scaled d-dimensional embedding coordinates matrix. Because of Eq (2), the embedding coordinates of the i^th^ cell of dataset A are obtained by the i^th^ row of *X*′, and those of the j^th^ cell of dataset B are obtained by the (n+j)^th^ row of *X*′.

Now, the dimensionality *d* needs to be determined. As the size of the original matrix *W* is *n* by *m*, its full rank is min (*n*, *m*) [15]. Thus, it is determined by Eq (6).

d  =min(n, m) (6)

### Agglomerative hierarchical clustering and evaluation of candidates

**Fig 2:**
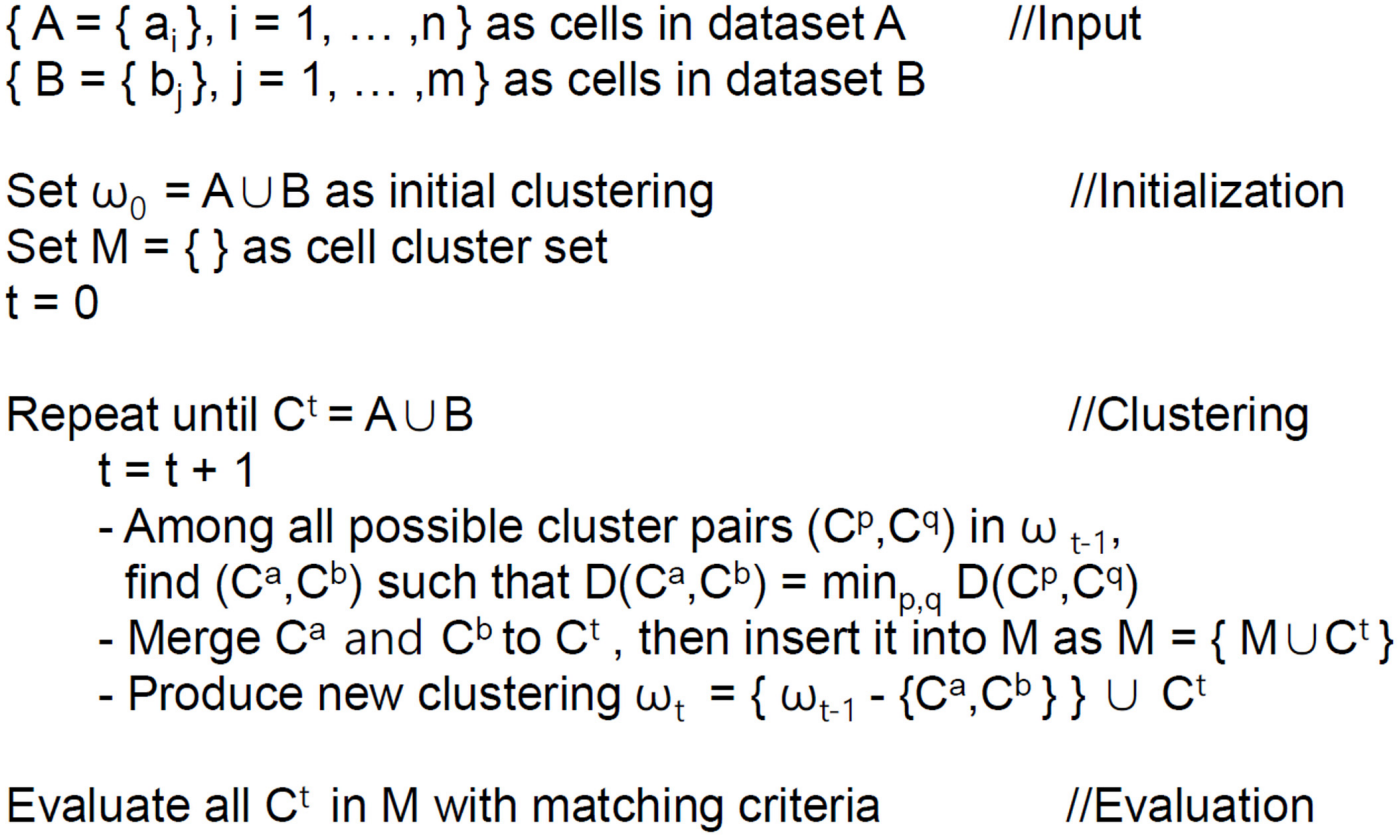
The pseudo-code of clustering method.

Among diverse clustering methods, an agglomerative hierarchical clustering is chosen because it expands initial cells one by one cell and searches CCPs. This process is similar to the buffer growing algorithm [7] as previously mentioned. Fig 2 presents a pseudo-code of the agglomerative hierarchical clustering and evaluation of candidate CCPs. Starting from initial cell clustering, the two most similar cell clusters C^a^ and C^b^ are identified and merged into one super-cell (cluster C^t^) as shown in Fig 1c. Now, new clustering is obtained by removing the two cell clusters C^a^ and C^b^ and inserting the super-cell cluster C^t^. This super-cell cluster is also inserted into a set of candidate cell clusters (M). These steps are repeated until all cells are merged into a single cell cluster.

To apply this clustering [18], the distance of two clusters *D*(*C*^*p*^, *C*^*q*^) needs to be measured. This distance is measured by the averaged distance of embedding coordinate vectors in the two clusters as shown in Eq (7) [19].

$$D\;\left(C^{p},\;C^{q}\right)\;=\;\frac{1}{\left|C^{p}\right|\;\left|C^{q}\right|}\sum_{a\in C^{p}}\sum_{b\in C^{q}}d\left(x_{a}^{p}\;,\;x_{b}^{q}\right)\;$$ (7)

where *C*^*p*^ and *C*^*q*^ are the p^th^ and q^th^ clusters, respectively, *d*(·) is the Euclidean distance function, |·| is the number of cells, and $x_{a}^{p}$, $x_{b}^{q}$ are embedding coordinate vectors of the a^th^ cell of *C*^*p*^ and the b^th^ cell of *C*^*q*^, respectively.

### ICP algorithm to detect corresponding vertex pairs

Given the cluster set M, each cell cluster is divided into two cell-sets according to their datasets, and their shape similarities are evaluated by the criterion of Eq (8) as shown in Fig 1d. Among the candidate CCPs obtained from the cell clusters, those with an *S*_1_(*A*^*l*^, *B*^*l*^) larger than a threshold *Th*_1_ are chosen for the CCPs.

$$S_{1}\;\left(\;A^{l}\;,\;B^{l}\;\right)\;=\;\frac{M\left(A^{l}\right)\;\cap \;f^{C}\left(\;M\left(\;B^{l}\;\right)\;\right)\;}{M\left(A^{l}\right)\;\cup f^{C}\left(\;M\left(\;B^{l}\;\right)\;\right)\;}\;\geq \;Th_{1}$$ (8)

where $A^{l}=\left\{\;a^{l}{}_{1},\cdots ,\;a^{l}{}_{\left|A^{l}\right|}\right\}$ and $B^{l}\;=\left\{\;b^{l}{}_{1},\;\cdots ,\;b^{l}{}_{\left|B^{l}\right|}\right\}$ are two cell-sets from the l^th^ cluster *C*^*l*^, and *M* and *f*^*c*^ present the functions that aggregate the disjointed cells into one super-cell and align the centroids of the two cell-sets *A*^*l*^ and *B*^*l*^, respectively.

**Fig 3:**
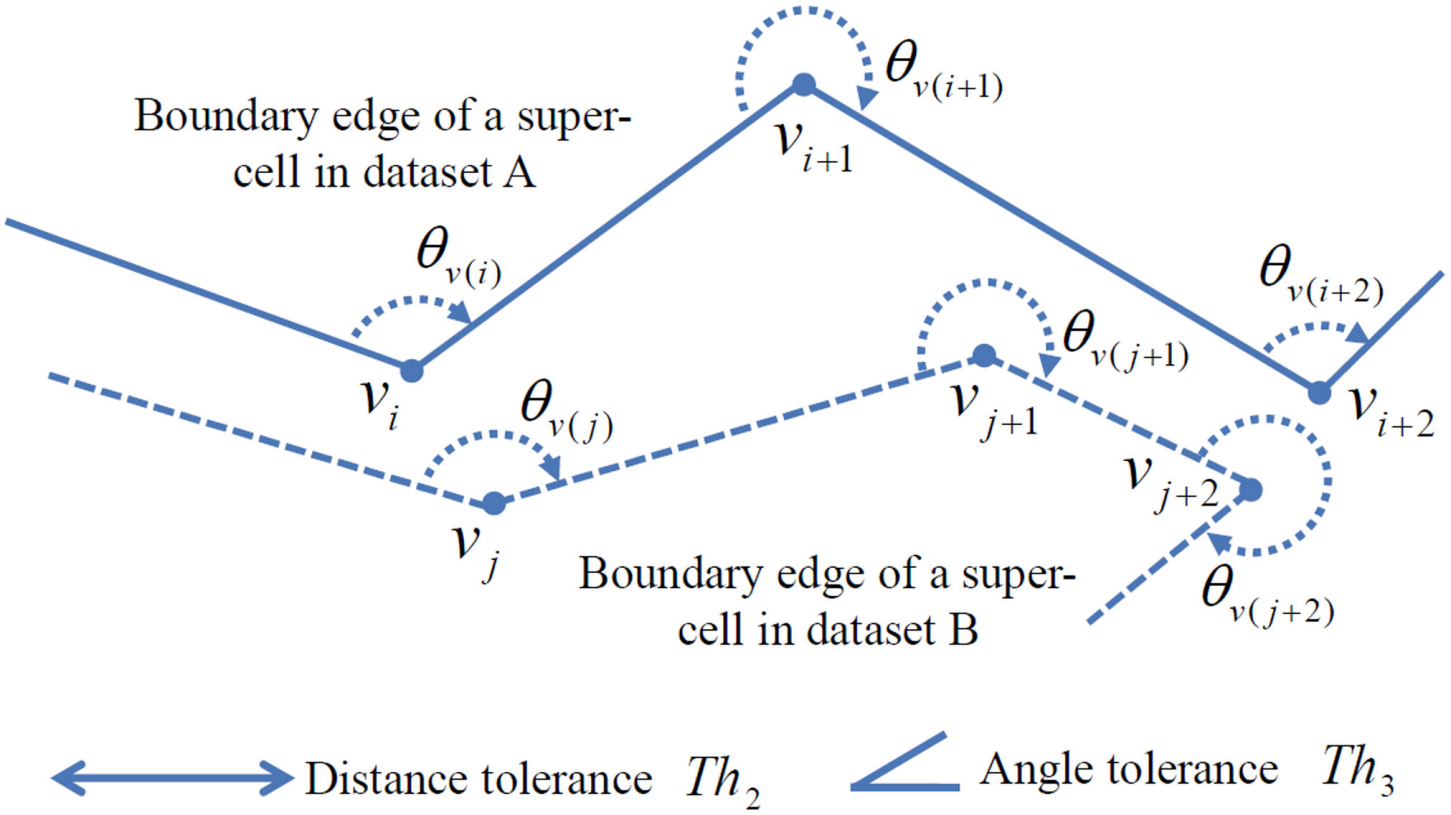
Vertex matching criteria. Vertices (*v*) and their interior angles (*θ*) of boundaries of super-cells in dataset A and B with distance and angle difference threshold.

Each cell-set of a CCP is aggregated into super-cells, and two vertex sets are extracted from the boundary edges of the each super-cell. Then the ICP algorithm [19] with a 6-parameter affine transformation model is applied to detect CVPs. This algorithm finds the closest vertex in one vertex set for each vertex in the other one, and then estimates a transformation model that best aligns the two vertex sets. This correspondence and transformation analysis is repeated until a termination condition. The original ICP algorithm only considers Euclidean distance because only coordinates are possible feature for the correspondence analysis. Meanwhile, the vertices in this study are vertices on the boundary edges of a super-cell. Thus, the coordinates and interior angle of a vertex can be used for the correspondence analysis as shown in Fig 3.

In this figure, when only closeness between coordinates is used to find CVPs, (*v*_(*i*)_, *v*_(*j*)_), (*v*_(*i*+1)_, *v*_(*j*+1)_) and (*v*_(*i*+2)_, *v*_(*j*+2)_) would be CVPs. However, the coordinates of *v*_(*i*+1)_ and *v*_(*j*+1)_ and the interior angles of *v*_(*i*+2)_ and *v*_(*j*+2)_ are too different each other though they are the closest pairs. To reject such erroneous pairs, we apply the distance and angle difference conditions of Eq (9).

$$d\left(v_{\left(i\right)},\;v_{\left(j\right)}\right)\;\leq \;Th_{\;2}\text{ and }\left|\;\theta_{v(i)}\;-\;\theta_{v(j)}\;\right|\;\leq \;Th_{\;3}$$ (9)

where *d*(*v*_(*i*)_, *v*_(*j*)_) is the Euclidean distance (m) between *v*_(*i*)_ and *v*_(*j*)_, and *θ*_*v*(*i*)_ and *θ*_*v*(*j*)_ are the interior angles (degree) of *v*_(*i*)_ and *v*_(*j*)_, respectively.

As the proposed method detects CCPs according to the hierarchical clustering, one vertex can be matched to several vertices of the other dataset. This is because the ICP algorithm is independently applied to each CCP. For example, cells 6 and 12 in Fig 1a constitute three CCPs of {5,6}:{10,11,12}, {2,3,4,5,6}:{8,9,10,11,12} and {1,2,3,4,5,6}:{7,8,9,10,11,12} according to the clustering result in Fig 1c. When the shapes of the cells’ bottom right corners are complicated, the corner’s detected CVPs for the three CCPs can be different. In this case, the final CVP of the corners is determined as the most frequently detected pairs. If more than one pairs are detected with the same largest frequency, the shortest pair is chosen for the final CVP.

### Map transformation with corresponding vertex pairs

Since the cell intersection degrees in Step 1 are affected by the positional discrepancy problem, erroneous CCPs can be obtained from the initial clustering result. To address this problem, we iterate the above CVP detection and a map transformation until a termination condition. Convectional affine or rigid transformation is not appropriate because their transformation averages local discrepancies equally over the entire coverage [20]. Thus we choose the smoothed thin plate spline transformation as shown in Eq (10) because of its ability to explain the global and local discrepancies [21].

$$f\left(x\;,y\right)\;=\;a_{1}\;+\;a_{x}\;x\;+\;a_{y}\;y\;+\;\sum_{\;k=1}^{\;P}\;w_{k}\;U\left(\;\left|\;(x_{k}\;,y_{k})\;-\;\left(\;x\;,\;y\;\right)\;\right|\;\right)$$ (10)

where (*x*_*k*_, *y*_*k*_) is the coordinates of the *k*^*th*^ CVP in the target dataset, *P* is the number of CVPs, *U*(*r*) is a radial function defined as *r*^2^log*r*^2^ where *r* is Euclidean distance between (*x*_*k*_, *y*_*k*_) and (*x*, *y*) as |(*x*_*k*_, *y*_*k*_)−(*x*, *y*)|. *a*_1_, *a*_*x*,_ *a*_*y*,_ *w*_*k*_ are the transformation coefficients obtained through the minimization of Eq (11) [21].

$$E\left(f\right)\;=\;\sum_{k=1}^{p}{\left|(x_{k}\;,y_{k})\;-\;f\left(x_{k}\;,y_{k}\right)\right|}^{2}\;+\;\lambda \;\iint \left[{\left(\frac{\partial^{2}f}{\partial \;x^{2}}\right)}^{2}\;+\;{\left(\frac{\partial^{2}f}{\partial \;x\;\partial \;y}\right)}^{2}\;+\;{\left(\frac{\partial^{2}f}{\partial \;y^{2}}\right)}^{2}\right]$$ (11)

This minimization problem can be resolved by applying the least square method to Eq (12) given CVPs in the form of *f*(*x*_*k*_, *y*_*k*_) = *v*_*k*_ = (*x*_*k*_′, *y*_*k*_′). Now, when *λ* is set to 0, the transformation function *f* exactly aligns the CVPs, whereas *λ* is set toward infinity, the function approach a hyperplane which is the least square fit of the CVPs [21]. The optimal value for *λ* can be obtained by the generalized cross validation method. However, it required significant computational burden. Thus, in this study, *λ* is heuristically set to the average entry value of *K* as $\;\left(\sum_{i,j}k_{i,j}\right)/{\left(size\;\left(K\right)\right)}^{2}$.

$$\left[\begin{array}{cc} K+\lambda \;I & P\\ P^{T} & 0 \end{array}\right]\;\left[\begin{array}{c} w\\ a \end{array}\right]\;=\;\left[\begin{array}{c} v\\ 0 \end{array}\right]$$ (12)

where *K* is a matrix whose entries are determined as *k*_*i*,*j*_ = *U*(|(*x*_*i*_, *y*_*i*_) − (*x*_*j*_, *y*_*j*_)|), *P* is a matrix whose k^th^ row is (1, *x*_*k*_, *y*_*k*_), *w* and *a* are column vectors formed from *w*_*i*_ and *a*_*i*_, respectively.

### Termination condition of iteration and final vertex matching

The iteration is terminated when the change ratio of the RMSE (m) of the CVPs is not meaningful. This stable condition is determined by Eq (13).

 |  RMSE i − RMSE i−1 |RMSE i    ≤   Th 4   and RMSE  i   ≤    Th 5 (13)

where *RMSE*_*i*_ is the RMSE of the CVPs detected at the i^th^ iteration.

Now the final CVPs are obtained as nearest vertex pairs between aligned two datasets. With the same idea of the distance condition of Eq (9), a tolerance distance is applied. As the 99% confidential interval of previously detected CVPs’ RMSE, the tolerance distance is set to 2.54 *RMSE*_*i*_.

## Results and Discussion

### Experimental data

**Fig 4:**
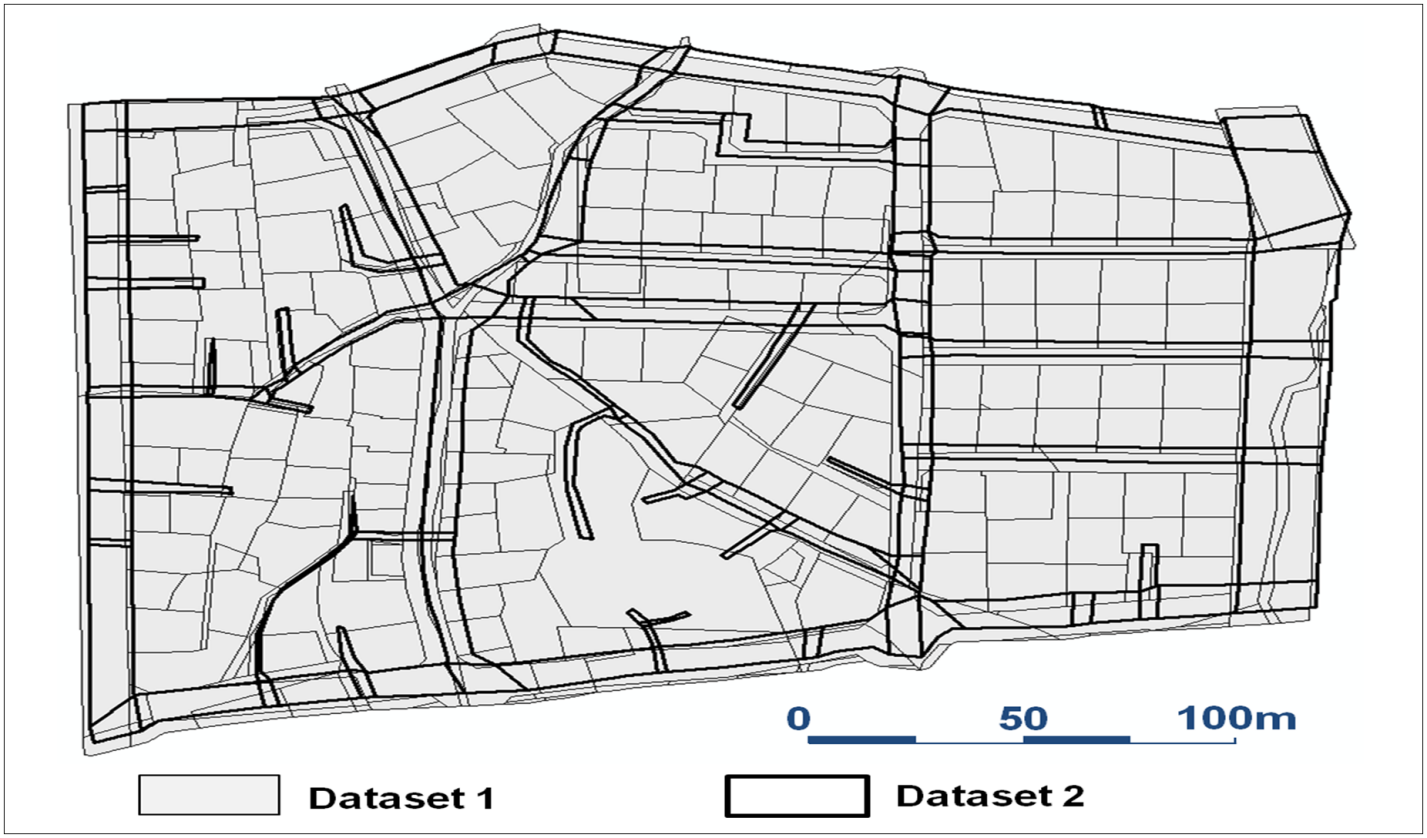
Two synthetic cell datasets for experiment. S1 and S2 Datasets are assumed as a cadastral map and a topographical map, respectively (Printed under a CC BY 4.0 license, with permission from Spatial Informatics & Systems Lab., Seoul National Univ.).

The proposed method was applied to two synthetic cell datasets assuming cadastral topographical maps (Fig 4). In general, the topographical map is the national base map with a high spatial quality. In the other hand, the cadastral map is created by joining and digitizing each legacy parcel-oriented map, which results in erratic and low spatial quality. Thus, the proposed method to detect the CVPs between the two maps can be an effective method to improve the spatial quality of the cadastral map. The topographical map has categorical layers such as transportation, building, hydrology, administration, elevation. However, the cadastral map is only related to land management, not the facilities over the ground. Thus, the layers of transportation and administration are chosen and spatially joined which makes the most area of the experimental topographical map has road and block cells as shown in dataset 2 of Fig 4.

The proposed method has five thresholds as shown in Table 1. *Th*_1_ controls the cell-set matching in terms of the shape similarity as shown in Eq (8). *Th*_2_ and *Th*_3_ impose the distance and angle constraints for CVPs as shown in Eq (9). Finally, *Th*_4_ and *Th*_5_ control the termination condition for the matching and transformation iteration.

**Table 1:** The thresholds of the proposed method.

*Th*_1_	*Th*_2_	*Th*_3_	*Th*_4_	*Th*_5_
0.83	5.8 (m)	10.2 (degree)	0.2	1.5 (m)

**Fig 5:**
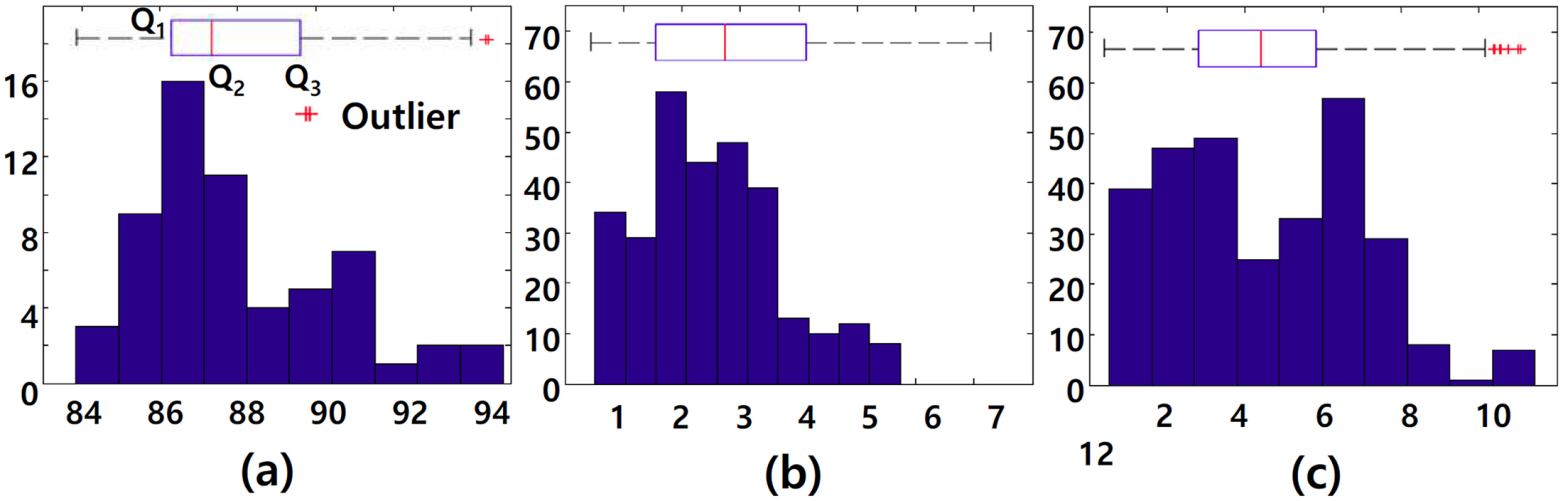
Thresholds training of the proposed method. The results of boxplot method to obtain the proposed method’s three thresholds. *Th*_1_(a), *Th*_2_(b), *Th*_3_(c) from manually chosen CCPs and CVPs in the training site in [4].

Among these thresholds, *Th*_1_, *Th*_2_ and *Th*_3_ should be determined as feasible lower or upper limits of the observed shape similarities of CCPs, distances and angle differences of CVPs in the training site. We applied the boxplot method [22] to training datasets of the central urban area of Suwon, Korea in [4]. In Fig 5, the bottom and top of a box represent the first quartile (*Q*_1_) and third quartile (Q3), and the band inside the box represent the median (*Q*_2_) of the observed geometries in the training site. Then, the upper and lower limits are determined as Eqs (14) and (15), respectively. In this study, *Th*_1_ is set as the lower limit of observed shape similarities of CCPs, and *Th*_2_ and *Th*_3_ as the upper limits of observed distances and angle differences of CVPs.

$$UL\;=\text{max }\left\{\;x\;|\;x\;<\;Q_{\;3}\;+\;1.5\cdot (Q_{\;3}\;-\;Q_{\;1})\;\right\}$$ (14)

$$LL\;=\text{min }\left\{\;x\;|\;x\;>\;Q_{\;1}\;-\;1.5\cdot (Q_{\;3}\;-\;Q_{\;1})\;\right\}$$ (15)

where *x* is an observed geometry in the training site. According to the above method as shown in Fig 5, the 3 thresholds were determined as 0.83, 5.8 (m) and 10.2 (degree), respectively. While, *Th*_4_ and *Th*_5_ were determined among several candidate values based on experimental results.

### Results and accuracy assessment

**Fig 6:**
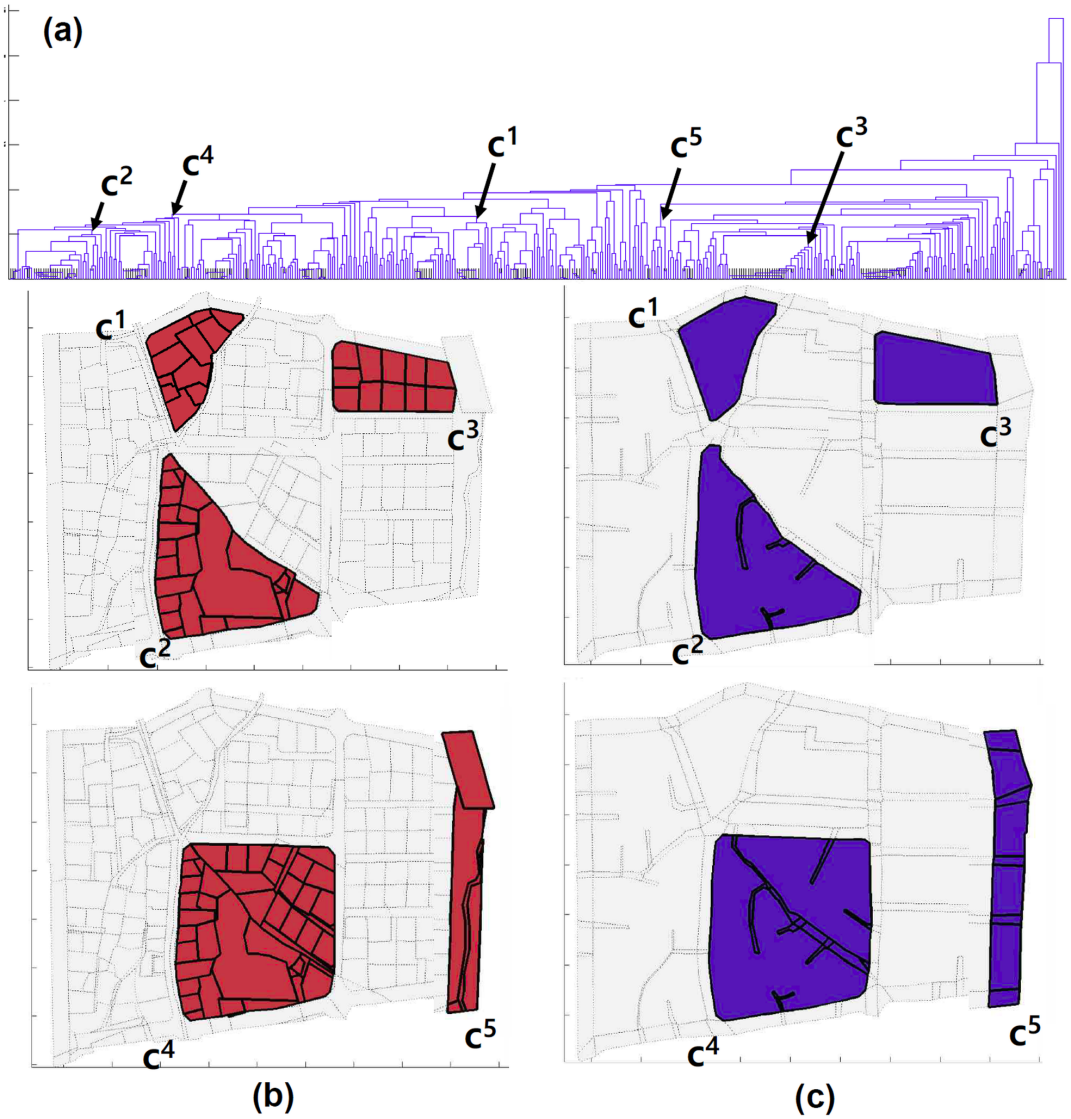
Agglomerative hierarchical co-clustering result of the synthetic datasets. Five detected CCPs in Fig 6 and the dendrogram of the first agglomerative hierarchical clustering (a) and the CCPs’ cell-sets in the dataset 1 (b) and dataset 2 (c) (Printed under a CC BY 4.0 license, with permission from Spatial Informatics & Systems Lab., Seoul National Univ.).

Similar to Fig 1c, the dendrogram of the first agglomerative hierarchical clustering of Fig 4 is obtained as Fig 6a. A dendrogram is a tree diagram in which the bottom row of nodes represents the individual cells of the two maps, and the remaining nodes represent the merging of their sub-nodes. Each cell cluster that corresponds to these nodes is divided into candidate corresponding cell-sets. Among these candidate pairs in Fig 6, five pairs (C^1^, C^2^, C^3^, C^4^ and C^5^) that satisfy the matching criteria are chosen for example. Due to the characteristics of this hierarchical clustering of the proposed method, CVPs of super-CCPs (e.g., C^4^) as well as those of sub-CCPs (e.g., C^2^) are obtained independently. This makes the proposed method choose reliable CVPs among many candidate CVPs independently detected from each CCP.

Table 2 shows RMSE of CVPs and its change ration at the i^th^ iteration. According to the terminal condition of Eq (13), the vertices of transformed the dataset 1 at the 5^th^ iteration are compared to those of the dataset 2 with the tolerance distance of 3.747 m (2.54 · 1.475m).

**Table 2:** RMSE of CVPs and its change ratio at the i^th^ iteration. S3 Dataset is the detected CVPs at 1^st^ iteration and S4 Dataset is the transformed S1 Dataset with S3 Dataset. S5 Dataset is the detected CVPs at 2^nd^ iteration and S6 Dataset is the transformed S4 Dataset with S5 Dataset. S7 Dataset is the detected CVPs at 3^rd^ iteration and S8 Dataset is the transformed S6 Dataset with S7 Dataset. S9 Dataset is the detected CVPs at 4^th^ iteration and S10 Dataset is the transformed S8 Dataset with S9 Dataset. S11 Dataset is the detected CVPs at 5^th^ iteration and S12 Dataset is the transformed S10 Dataset with S11 Dataset.

Iteration	1^st^	2^nd^	3^rd^	4^th^	5^th^
RMSE (m)	2.741	1.686	1.711	1.603	1.475
Change ratio of RMSE	-	0.626	0.015	0.067	0.087

**Fig 7:**
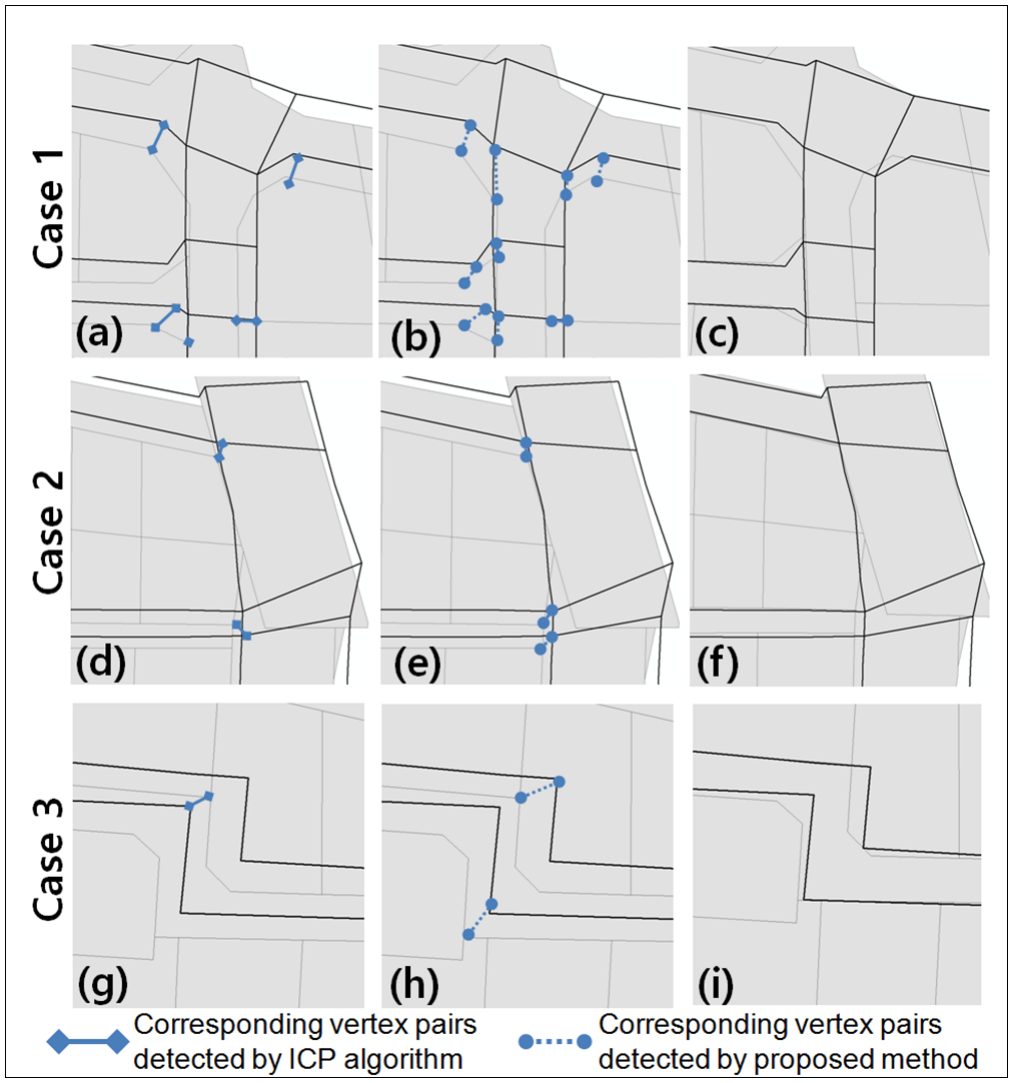
Comparison of the detection results. Results by applying the ICP algorithm(a, d, g) and those by the proposed method (b, e, h); (c), (f) and (i) show the transformed dataset 1 at the final iteration and the dataset 2 (Printed under a CC BY license 4.0, with permission from Spatial Informatics & Systems Lab., Seoul National Univ.).

Fig 7 compares three cases of the detected CVPs by applying the ICP algorithm with the same conditions of Eq (9). Huh et al. [4] shrunk all cells of the maps by $Th{\;}_{2}/\sqrt{2}$, and then searched connected cell-sets along the shrunk cells’ intersection relationship. However, Huh et al. [4] assumed cells whose sizes are sufficiently larger than the shrinking tolerance. Thus, given narrow street cells such as Fig 4, they can be collapsed and not be used for constituting CCPs. To prevent this problem in this study, a cell’s centreline is alternatively used for its shrunk cell. Then CPRs and their CVPs are detected similar to the proposed method.

These cases show the effect of reducing the uneven positional discrepancies by the iterative matching and transformation process of the proposed method. In the first case, the previous and the proposed methods detect seven and nine CVPs, respectively. All the CVPs connect proper positions between two maps except one common CVP at the right bottom area of Fig 7a and 7b. The proposed method find two more CVPs because the proposed method’s iterative matching and transformation process gradually reduces the aforementioned discrepancies, and thus makes the corresponding geometries between the maps closer and more similar each other as shown in Fig 7c. In the second case, the bottom CVP in Fig 7d connects vertices from different cells; meanwhile the proposed method finds proper ones as shown in Fig 7e. This is because the narrow street cell bridges upper and bottom block cells and presents erroneously a large CCP of Huh et al. [4]. Thus CVPs along the narrow street cannot be properly detected. This problem also occurs in the third case as shown in Fig 7g. However, the proposed method detects proper ones as shown in Fig 7e and 7h because the both maps are well locally aligned as shown in Fig 7f and 7i.

To statistically compare the performance of the proposed method with that of the ICP algorithm, we used three types of measures: precision, recall and Fmeasure (Eq 16).

$$\text{ Fmeasure}=\text{ 2 }\times \;\frac{\text{precision }\times \text{ recall}}{\text{precision }+\text{ recall}}$$ (16)

where precision is the ratio of the number of true CVP detections to that of all of the detected CVPs and recall is the ratio of the number of true CVP detections to that of the manually detected reference pairs. We applied the two methods for the test site of Fig 4. As shown in Table 3, the precision and recall of the proposed method were 0.85 and 0.82, respectively; those of the ICP algorithm were 0.63 and 0.38, respectively. Thus, the Fmeasures of the two methods were 0.84 and 0.48, respectively. The precisions of the both methods were similar each other, while the recall of the proposed method was higher. This means that the proposed method detects more CVPs than the previous method with similar probability of false detection as shown in Fig 7. This improvement was obtained through the proposed method’s iterative CCP and CVP matching and transformation process which makes the corresponding geometries between the maps closer and more similar each other. The CCP detection is a local bottom-up search which makes the detection insensitive to the uneven positional discrepancies. Moreover, CCPs have a hierarchical structure which means that CVPs of super-CCPs as well as those of sub-CCPs are obtained independently. Thus, more accurate and plausible ones can be chosen from these abundant candidate CVPs, which leads to an improvement for CVP detection.

**Table 3:** The statistical evaluation of the proposed method and the ICP algorithm for datasets in Fig 4. S13 Dataset is the detected CVPs by the ICP algorithm and S14 Dataset is the transformed S2 Dataset with S13 Dataset. S15 Dataset is manually detected corresponding point pairs between S10 Dataset and S1 Dataset for statistical evaluation.

	Precision	Recall	Fmeasure
Proposed method	0.85(181/212)	0.82(181/221)	0.84
ICP algorithm	0.63(85/135)	0.38 (85/221)	0.48

## Conclusions

The rapid development of location-based services on web portals and mobile devices, has led diverse organisations to construct spatial datasets with their own data acquisition methods and spatial quality standards. To conflate these datasets, CVPs need to be detected to reduce the positional discrepancies. However, given complicated M:N cell-set pairs and uneven positional discrepancies between cell datasets, a new method to detect abundant and accurate CVPs is necessary.

To address this problem, the proposed method applies agglomerative hierarchical co-clustering to detect CCPs, and then detect CVPs with the ICP algorithm for each CCP. The basic idea of the proposed method is similar to the buffer growing algorithm because the both methods iteratively expand an object-set pair by one object from either of two datasets until a corresponding pair is obtained. To determine the priority for the expansion in this study, cell intersection degrees are applied. However, these degrees, especially for small cells, are easily affected by the aforementioned discrepancies. To address this problem, the above CVPs detection and map transformation are iterated to make the corresponding geometries between the maps closer and more similar each other. Then final CVPs are obtained as nearest vertex pairs within a tolerance distance.

The proposed method was applied to synthetic datasets. The experiments indicated that the performance of the proposed method was superior to that of the ICP algorithm. The precision and recall of the proposed method were 0.85 and 0.82, respectively. And those of the ICP algorithm were 0.63 and 0.38, respectively. Therefore, the proposed method can detect more abundant true CVPs. This improvement was obtained by the following characteristics. The CCP detection is a local bottom-up search which makes the cell matching insensitive to the uneven positional discrepancies between datasets. Moreover, these CCPs have a hierarchical structure which means that CVPs of super-CCPs as well as their sub-CCPs are obtained by independent vertex matching. Thus, accurate and abundant CVPs which are insensitive to the uneven positional discrepancies between datasets can be obtained.

## Supporting Information

S1 Dataset Synthetic dataset 1. (ZIP)

S2 Dataset Synthetic dataset 2. (ZIP)

S3 Dataset Corresponding vertex pairs between S1 and S2 Datasets at the 1^st^ iteration. (ZIP)

S4 Dataset Transformed S1 Dataset with S3 Dataset. (ZIP)

S5 Dataset Corresponding vertex pairs between S4 and S2 Datasets at the 2^nd^ iteration. (ZIP)

S6 Dataset Transformed S4 Dataset with S5 Dataset. (ZIP)

S7 Dataset Corresponding vertex pairs between S6 and S2 Datasets at the 3^rd^ iteration. (ZIP)

S8 Dataset Transformed S6 Dataset with S7 Dataset. (ZIP)

S9 Dataset Corresponding vertex pairs between S8 and S2 Datasets at the 4^th^ iteration. (ZIP)

S10 Dataset Transformed S8 Dataset with S9 Dataset. (ZIP)

S11 Dataset Corresponding vertex pairs between S10 and S2 Datasets at the 5^th^ iteration. (ZIP)

S12 Dataset Final corresponding point pairs between S10 and S2 Datasets. (ZIP)

S13 Dataset Corresponding vertex pairs between S1 and S2 Datasets by the previous ICP algorithm. (ZIP)

S14 Dataset Transformed S1 Dataset with S13 Dataset. (ZIP)

S15 Dataset Manually detected corresponding vertex pairs between S10 and S1 Datasets. (ZIP)
